# The Secretome Derived From Human Umbilical Cord Mesenchymal Stem Cells Improves Skin Photoaging by Enhancing Mitophagy to Inhibit the cGAS‐STING Pathway

**DOI:** 10.1111/acel.70701

**Published:** 2026-09-11

**Authors:** Tingting Tang, Meifen Lin, Jingjing Yang, Xinyu Yang, Xunhong Xu, Xuan Wang, Yarui Zhang, Qimei Chen, Shan Zhao, Chenhui Guo, Hao Zhang, Min Zhang, Lin Zhang, Xueer Wang

**Affiliations:** ^1^ Department of Histology and Embryology, School of Basic Medical Sciences Southern Medical University Guangzhou China; ^2^ Department of Pathology Wuping County Hospital Longyan Fujian China; ^3^ School of Public Health Southern Medical University Guangzhou China; ^4^ Guangdong Provincial Key Laboratory of Construction and Detection in Tissue Engineering Southern Medical University Guangzhou China; ^5^ NMPA Center for Innovation and Research in Regulatory Science Guangzhou China; ^6^ Key Laboratory of Functional Proteomics of Guangdong Province, School of Basic Medical Sciences Southern Medical University Guangzhou China

**Keywords:** autophagy, cell‐free therapy, cGAS‐STING, mesenchymal stem cell, mitophagy, photoaging, secretome, skin inflammation

## Abstract

Ultraviolet (UV)‐induced skin photoaging is driven by cellular senescence and chronic inflammation. Although the secretome (SCT) derived from human umbilical cord mesenchymal stem cells (hUC‐MSC SCT) exhibits regenerative potential, its role in combating photoaging remains to be elucidated. In this study, we show that SCT markedly ameliorates photoaging in UV‐exposed mice, restoring epidermal barrier function, dermal collagen integrity, senescence‐associated markers, and systemic inflammatory profiles. In UVB‐irradiated human immortalized keratinocytes (HaCaT), SCT enhanced cellular viability, proliferation, and migration while alleviating cellular senescence. Mechanistically, SCT promoted mitophagy, as indicated by clearance of mitochondrial proteins (TOM20, TIM23, HSP60), reversal of p62 accumulation, and restoration of LC3B‐II flux. By eliminating impaired mitochondria, SCT reduced cytosolic mtDNA leakage, thereby suppressing the overactivation of the cGAS‐STING pathway and its downstream pro‐inflammatory cytokines IL‐6, IL‐8, and IFN‐β. Importantly, these protective effects were abolished by the mitophagy inhibitor Mdivi‐1, whereas the STING inhibitor H151 effectively reversed the Mdivi‐1‐induced loss of protection, confirming that SCT acts through the hierarchical mitophagy–cGAS‐STING axis. Together, our findings establish SCT as a promising cell‐free therapeutic strategy for photoaging and highlight the mitophagy–cGAS‐STING axis as a critical nexus linking mitochondrial quality control to sterile inflammation in aging tissues.

## Graphical abstract

HUC‐MSC‐derived SCT alleviates UV‐induced skin photoaging. It acts by enhancing mitophagy to clear damaged mitochondria, thereby suppressing the pro‐inflammatory cGAS‐STING pathway. This work identifies SCT as a novel cell‐free therapy targeting mitochondrial quality control to counteract sterile inflammation in aging.

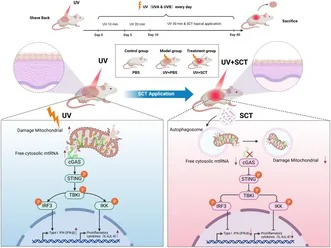

## 1 Introduction

Skin photoaging is an extrinsic aging process primarily induced by chronic ultraviolet (UV) radiation exposure (Xue et al. 2022; Jiang et al. 2025; Cavinato and Jansen‐Dürr 2017). UV radiation is classified into three categories based on wavelength: UVC (100–280 nm), UVB (280–320 nm), and UVA (320–400 nm) (Zhang, Xiao, et al. 2024). While most UVC is absorbed by the ozone layer, both UVA (approximately 95%) and UVB (approximately 5%) reach the Earth's surface (Lopes et al. 2022). UVB is predominantly absorbed by the epidermis and directly damages keratinocytes, while UVA penetrates deeper into the dermis to affect fibroblasts and the extracellular matrix homeostasis. Both wavelengths contribute synergistically to the clinical manifestations of photoaging (Guan et al. 2021). The clinical manifestations of photoaging include skin roughness, laxity, wrinkles, telangiectasia, and pigmentation disorders (Dorf and Maciejczyk 2024). In severe cases, it can progress to precancerous lesions such as actinic keratosis or even cutaneous malignancies like squamous cell carcinoma, significantly impacting patients' physical and psychological health (Sun et al. 2025; Cavinato et al. 2024). At the cellular level, photoaging is characterized by the accumulation of senescent cells, which exhibit elevated expression of cell‐cycle inhibitors such as p16 and p21, alongside a decline in the nuclear envelope protein Lamin B1 (Zhang, Xiao, et al. 2024; Zhang, Wang, et al. 2024). Epidemiological studies have shown that the incidence of photoaging is closely associated with environmental UV intensity, individual sun protection behaviors, and genetic susceptibility, with outdoor workers and high‐altitude residents exhibiting significantly higher prevalence rates than the general population (Diehl et al. 2024; Grandahl et al. 2018; Rocholl et al. 2021). Current clinical interventions include sunscreens, topical retinoids (e.g., retinol), antioxidants (e.g., vitamin C and niacinamide), and energy‐based devices (e.g., fractional lasers and intense pulsed light). However, these approaches present notable limitations: sunscreen efficacy depends heavily on user compliance and does not provide complete UV protection; retinoids frequently cause skin irritation, including erythema and desquamation; antioxidants suffer from low transdermal efficiency and poor stability; and photoelectric therapies are often costly and may lead to adverse effects such as post‐inflammatory hyperpigmentation (Toor et al. 2025; Zhang, Li, et al. 2025; Shen et al. 2025; Berry et al. 2023; Zhu et al. 2025; Passeron et al. 2021; Geisler et al. 2021). Consequently, the development of more efficient, safe, and user‐friendly therapeutic strategies remains a critical research direction in the field of skin photoaging.

Mesenchymal stem cells (MSCs) are adult stem cells possessing self‐renewal and multi‐directional differentiation capabilities, which have demonstrated significant therapeutic potential in various disease models (Lee et al. 2026; Zhang, Xie, et al. 2025; Lit et al. 2025). Among them, human umbilical cord‐derived MSCs (hUC‐MSCs) have garnered considerable attention in clinical applications due to their accessibility, high proliferative capacity, absence of ethical concerns, and non‐invasive procurement from donors (Dong et al. 2018). Studies indicate that the therapeutic effects of MSCs primarily stem from their paracrine functions rather than direct differentiation and replacement (Li, Tao, et al. 2025). The secretome (SCT) refers to the entirety of bioactive factors secreted or released by cells into the extracellular environment, including proteins (such as cytokines and growth factors), nucleic acids (e.g., miRNAs, lncRNAs), lipid metabolites, and extracellular vesicles (e.g., exosomes and microvesicles) (Zhang, Wang, et al. 2025). These components mediate intercellular communication and participate in the regulation of tissue repair, immune homeostasis, and disease progression through paracrine or endocrine mechanisms (Trigo et al. 2025). In recent years, the MSC‐derived SCT has emerged as a promising “cell‐free” therapeutic strategy due to its notable immunomodulatory and tissue‐repair functions. Research has shown that the MSC‐derived SCT exhibits therapeutic effects in multiple disease models, such as repairing meniscus injury and promoting the healing of diabetic skin wounds (Trigo et al. 2025; Chen, Lai, et al. 2025). Our recent study demonstrated that the secretome derived from hUC‐MSCs promotes hair growth and cycle transition by enhancing methylthioadenosine synthesis, a process mediated through the PI3K/AKT/mTOR signaling pathway (Zhang, Wang, et al. 2025). However, the application of the SCT in dermatology, particularly for photoaging, remains in its early stages; this is largely due to the fact that the specific molecular pathways mediating its anti‐photoaging effects are still undefined, representing a critical knowledge gap that impedes clinical translation.

Mitophagy, a selective autophagy process responsible for clearing damaged mitochondria, serves as a critical quality control mechanism and plays a key regulatory role in skin photoaging (Cavinato et al. 2024; Lu et al. 2025). Mitochondria are central to cellular metabolism and signaling, but they are also a primary source of endogenous ROS and are highly vulnerable to UV‐induced damage. Chronic UV exposure can lead to mutations in mitochondrial DNA (mtDNA), impaired respiratory chain function, and excessive ROS production, creating a vicious cycle of oxidative damage that promotes cellular senescence and apoptosis (Guerrero‐Navarro et al. 2024). Under physiological conditions, mitophagy efficiently removes these dysfunctional mitochondria, thereby preserving cellular health and function. However, emerging evidence suggests that mitophagy activity is notably compromised during the photoaging process, leading to the accumulation of damaged mitochondria and exacerbating cellular dysfunction (Chen, Lin, et al. 2025; Sreedhar et al. 2020).

Concurrently, the cyclic GMP‐AMP synthase (cGAS)‐stimulator of interferon genes (STING) pathway has been recognized as a key mediator of sterile inflammation in the aging process (Mun et al. 2025). The cGAS protein acts as a cytosolic DNA sensor. When damaged mitochondria release their mtDNA into the cytoplasm, cGAS recognizes this misplaced self‐DNA and synthesizes the second messenger cGAMP. This, in turn, activates the adaptor protein STING, triggering a downstream signaling cascade involving TBK1 and IRF3. This cascade culminates in the production of Type I interferons and other pro‐inflammatory cytokines, including IL‐6, IL‐8, and IFN‐β, driving a state of chronic, low‐grade inflammation often referred to as “inflammaging” (Alipour‐Khezri et al. 2025). Accumulating evidence indicates that sustained activation of the cGAS‐STING pathway is a crucial driver of tissue aging and age‐related pathologies (Wang, Yu, et al. 2025; Li, Cai, et al. 2025). Intriguingly, a functional link between mitophagy and the cGAS‐STING pathway is beginning to emerge. Studies in the context of neurodegeneration have shown that by clearing damaged mitochondria, efficient mitophagy prevents the release of mtDNA into the cytosol, thereby suppressing aberrant cGAS‐STING activation and its pro‐inflammatory consequences (Su et al. 2025; Meng et al. 2026; Wang, Wu, et al. 2025). This positions the mitophagy–cGAS‐STING axis as a central regulatory node in organ aging. However, whether this axis plays a central and therapeutically targetable role in skin photoaging, particularly in the context of MSC‐derived SCT intervention, remains to be fully elucidated.

Therefore, this study was designed to address these knowledge gaps. We hypothesize that the therapeutic effects of the SCT on skin photoaging are mediated, at least in part, by the restoration of deficient mitophagy. We further postulate that by enhancing the clearance of damaged mitochondria, the SCT can suppress the aberrant activation of the pro‐inflammatory cGAS‐STING signaling pathway. To this end, we employed pharmacological modulators, including the mitophagy inhibitor Mdivi‐1 and the STING inhibitor H151, to dissect the causal hierarchy within this signaling axis. The primary objective of this research is to systematically investigate the efficacy of SCT in an in vivo photoaging mouse model and in vitro UVB‐stimulated cell senescence model, and to clarify the underlying molecular mechanisms, with a specific focus on dissecting the functional interplay within the mitophagy–cGAS‐STING signaling axis. The findings from this study are expected to provide a novel mechanistic basis for the anti‐photoaging effects of the MSC‐derived SCT and may pave the way for the development of a new class of cell‐free biotherapeutics for skin rejuvenation.

## 2 Results

### 2.1 SCT Ameliorates UV‐Induced Skin Photoaging in Mice

**FIGURE 1:**
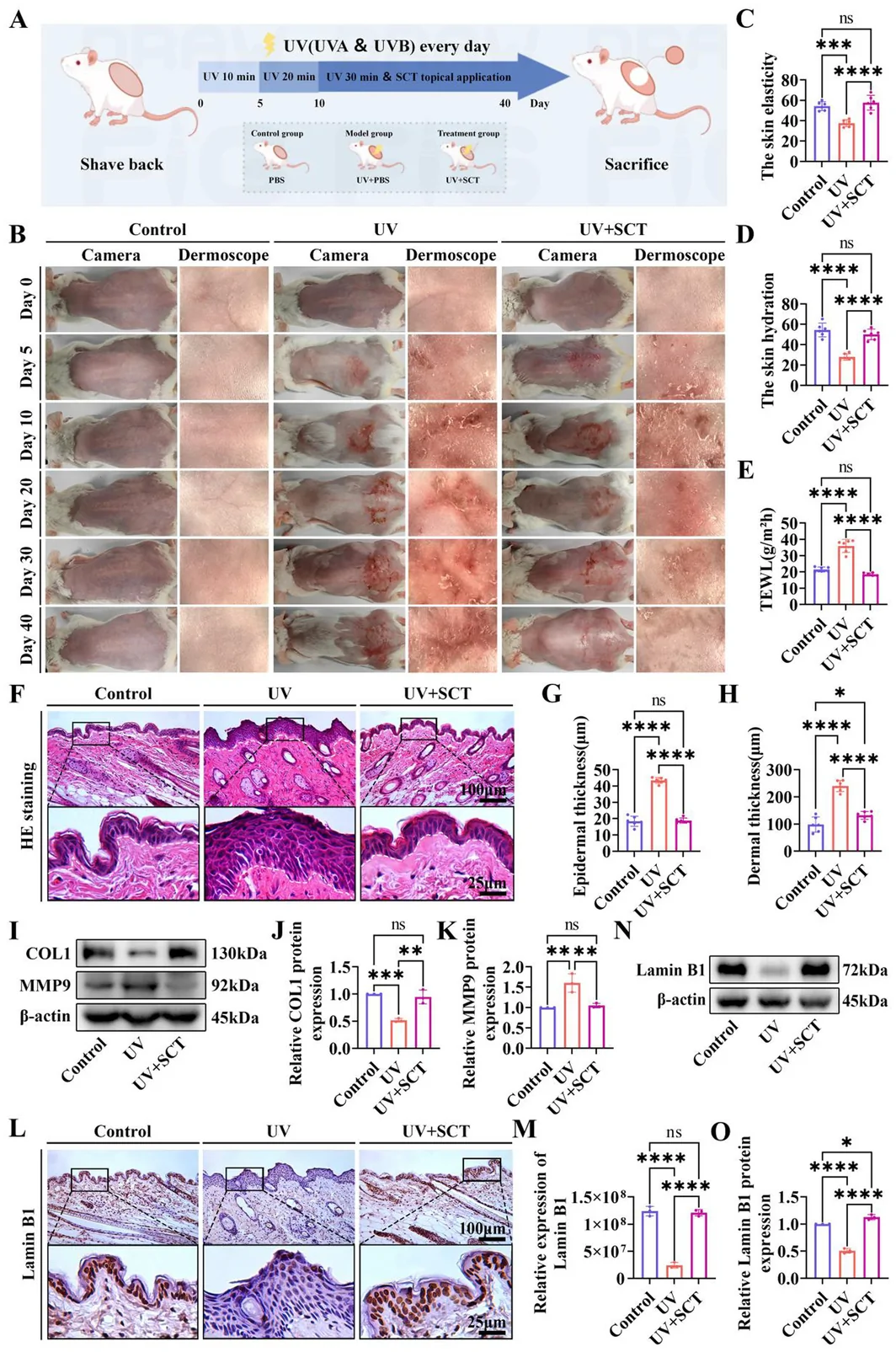
Histological analysis of the dorsal skin in KM mice after UV irradiation and SCT treatment. (A) Schematic description of the establishment and treatment of the photoaging model (Created with Figdraw.com). (B) Representative images of mouse dorsal skin. From left to right: control group, UV‐exposed groups treated with PBS, and SCT. (C) Skin elasticity, *n* = 6. (D) Epidermal hydration, *n* = 6. (E) Skin barrier function was detected by transepidermal water loss (TEWL), *n* = 6. (F) Representative images of HE staining (scale bar, 100 μm). (G) Epidermal thickness analysis, *n* = 6. (H) Dermal thickness analysis, *n* = 6. (I–K) Western blotting was used to measure COL1 and MMP9 expression in skin tissues, *n* = 3. (L, M) Immunohistochemical staining and statistics of relative levels of Lamin B1, *n* = 3 (scale bar, 100 μm). (N, O) Western blotting was used to measure Lamin B1 expression in skin tissues, *n* = 3. *p < 0.05, **p < 0.01, ***p < 0.001, and ****p < 0.0001.

To evaluate the effect of SCT on skin photoaging, a UV‐induced photoaging mouse model was established according to previously described methods (Figure 1A) (Lin et al. 2023). Dorsal skin images were captured and skin conditions were assessed on Days 0, 5, 10, 20, 30, and 40 following UV irradiation. Mice subjected to UV exposure exhibited marked signs of chronic photoaging, including significant skin thickening, leather‐like appearance, erythema, and desquamation, indicating successful model establishment. Meanwhile, SCT treatment markedly ameliorated these photoaging features (Figure 1B). Assessment of skin physiological parameters revealed that, compared with the control group, UV‐exposed mice showed a significant reduction in skin elasticity and epidermal hydration, along with a pronounced increase in transepidermal water loss. These changes were effectively reversed by SCT treatment (Figure 1C–E).

H&E staining revealed that SCT significantly attenuated UV‐induced epidermal hyperplasia and also alleviated the increase in dermal thickness (Figure 1F–H). Having established the effects of SCT on epidermal barrier function, we next examined dermal connective tissue, given that collagen and extracellular matrix remodeling are primary drivers of UV‐induced photoaging. Masson's trichrome staining showed that chronic UV irradiation caused prominent fragmentation and loss of dermal collagen fibers, whereas SCT treatment preserved collagen fiber integrity and significantly increased total dermal collagen content (Figure S1A,B). Immunohistochemistry further revealed that UV exposure downregulated COL1 and COL3 while upregulating MMP1, MMP3, and MMP9; SCT supplementation restored COL1 and COL3 levels and attenuated the UV‐induced upregulation of these MMPs (Figure S1C,D). These findings were corroborated by Western blot analysis of skin homogenates, which confirmed that UV‐induced downregulation of COL1 and upregulation of MMP9 were normalized by SCT treatment (Figure 1I–K). Collectively, these data suggest that SCT may exert its protective effects on dermal collagen homeostasis, at least in part, through modulating matrix metalloproteinase activity, thereby counteracting UV‐induced collagen degradation.

We also included a SCT‐only group (without UV exposure) to assess whether SCT affects normal skin homeostasis in 5‐month‐old KM mice. Under non‐stressed physiological conditions, SCT treatment induced modest but statistically significant improvements in barrier function parameters, including epidermal hydration and transepidermal water loss (Figure S2A–C), suggesting that SCT contributes to skin moisturization and barrier maintenance under homeostatic conditions. However, H&E staining revealed no detectable epidermal or dermal thickening (Figure S2D–F), and Masson's trichrome staining along with immunohistochemical analyses of COL1, COL3, and MMP1/3/9 showed no significant differences between the control and SCT‐only groups (Figure S1). These findings indicate that SCT does not constitutively alter intact dermal extracellular matrix turnover. Taken together, these results suggest that SCT exhibits a context‐dependent dual role: under normal physiological conditions, it supports hydration and barrier integrity without perturbing skin homeostasis; under UV‐induced inflammatory and oxidative stress, it actively exerts reparative effects to counteract tissue damage. This stress‐responsive protective profile underscores the safety and specificity of SCT application.

To further validate the anti‐photoaging effects of SCT, we examined senescence‐associated markers by immunohistochemistry and western blotting. Immunohistochemistry showed that Lamin B1 expression was significantly downregulated in the epidermis after UV irradiation and effectively restored by SCT treatment (Figure 1L,M); these findings were confirmed by Western blot analysis (Figure 1N,O). We further measured two additional senescence biomarkers, p21 and p16. Chronic UV exposure significantly elevated epidermal p21 and p16 levels, whereas SCT application markedly suppressed this upregulation (Figure S3A,B).

Collectively, these findings demonstrate that SCT ameliorates UV‐induced skin photoaging by restoring both epidermal barrier integrity and dermal collagen architecture.

### 2.2 SCT Alleviates UVB‐Induced Photoaging in HaCaT In Vitro

**FIGURE 2:**
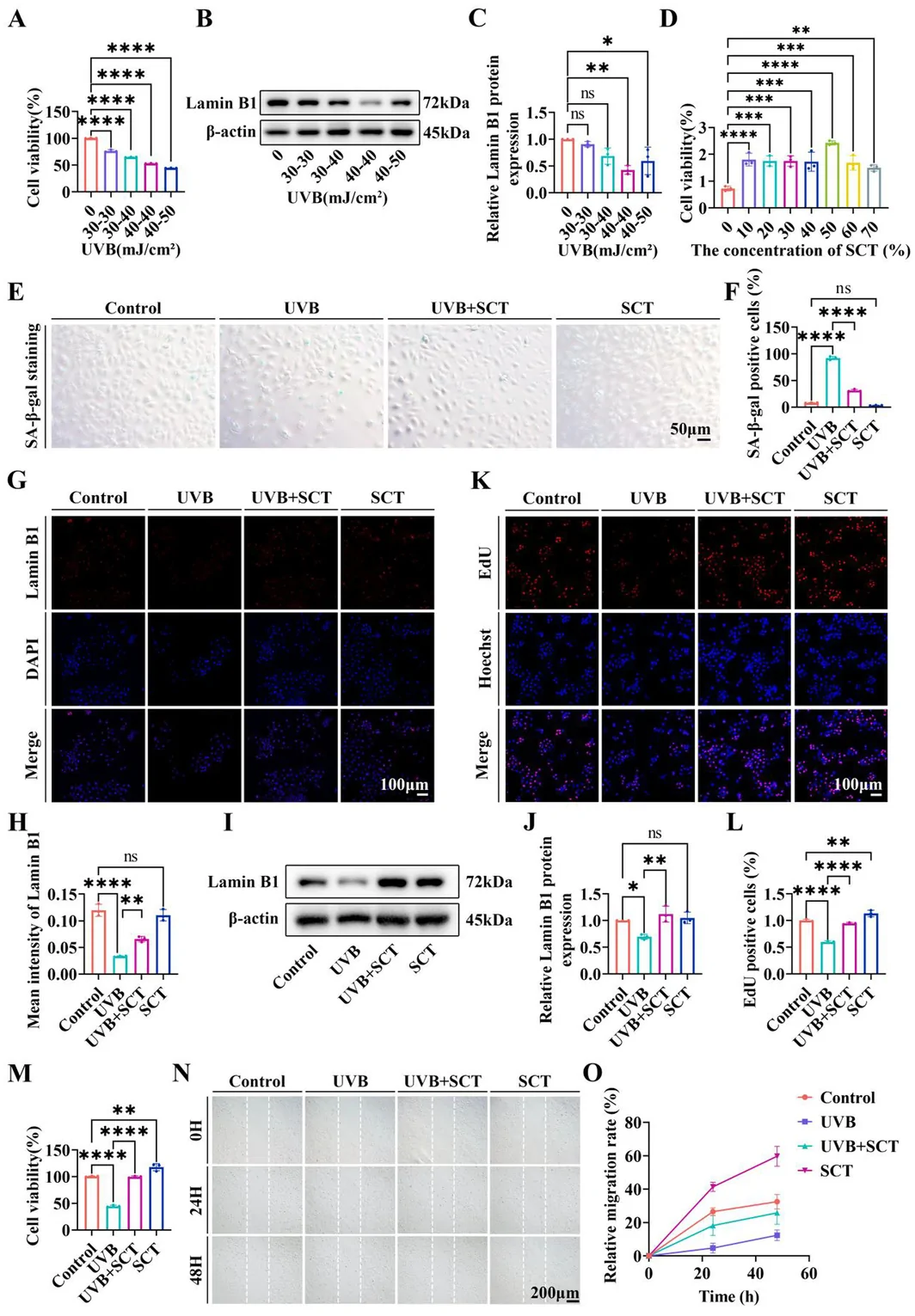
SCT mitigates photoaging in HaCaT cells in vitro. (A) Cell viability was measured using the CCK‐8 assay in HaCaT cells exposed to increasing doses of UVB radiation, *n* = 3. (B, C) Western blotting was used to measure Lamin B1 expression in HaCaT cells exposed to increasing doses of UVB radiation, *n* = 3. (D) CCK‐8 assay evaluating HaCaT cells' viability under various SCT concentrations, *n* = 3. (E) Representative images of SA‐β‐gal staining in HaCaT cells (scale bar, 50 μm). (F) SA‐β‐gal‐positive cells in HaCaT cells, *n* = 3. (G, H) Immunofluorescence staining and statistics of relative levels of Lamin B1 in HaCaT cells, *n* = 3 (scale bar, 100 μm). (I, J) Western blotting was used to measure Lamin B1 expression in HaCaT cells, *n* = 3. (K, L) HaCaT cells' proliferation in each group was determined by EdU staining, *n* = 3 (scale bar, 100 μm). (M) HaCaT cells' viability in each group was detected by CCK‐8 assays, *n* = 3. (N) Representative images of the migration assay were taken at the indicated time points (scale bar, 200 μm). (O) Quantitation of migration assays of HaCaT cells, *n* = 3. *p < 0.05, **p < 0.01, ***p < 0.001, and ****p < 0.0001.

After confirming the ameliorative effect of SCT on skin photoaging in KM mice, we further investigated its underlying mechanisms using human immortalized keratinocytes (HaCaT) as an in vitro model. HaCaT cells effectively replicate the biological characteristics of epidermal keratinocytes, which are direct targets of UVB radiation (Zhang, Xiao, et al. 2024). To establish an in vitro photoaging model, HaCaT cells were exposed to UVB at varying total doses delivered in two low‐dose fractions. CCK‐8 assays revealed a dose‐dependent reduction in cell viability following UVB irradiation (Figure 2A). Western blot analysis further demonstrated that UVB significantly downregulated the expression of the senescence‐associated protein Lamin B1, with the most pronounced aging phenotype observed at a total dose of 80 mJ/cm^2^, which was consequently selected for subsequent experiments (Figure 2B,C).

We next evaluated the effect of SCT on UVB‐induced cellular senescence. SCT concentrations ranging from 0% to 70% were evaluated using a CCK‐8 assay, with the highest cell viability observed at 50% SCT, with the most effective response observed at 50% SCT; this concentration was therefore used in further studies (Figure 2D). SA‐β‐gal staining indicated that UVB irradiation led to an accumulation of β‐galactosidase‐positive cells, which was significantly attenuated by SCT treatment (Figure 2E,F). Consistent with this, both immunofluorescence and Western blot analyses confirmed that SCT effectively restored UVB‐induced downregulation of Lamin B1 expression (Figure 2G–J). In addition, EdU staining and CCK‐8 assays showed that SCT rescued the UVB‐induced suppression of cell proliferation and viability (Figure 2K–M), while cell migration assays indicated that SCT promoted the migration capacity of HaCaT cells following UVB injury (Figure 2N,O).

Collectively, these in vitro results verify the robust anti‐senescence activity of SCT in UVB‐irradiated keratinocytes, providing a solid basis for further mechanistic exploration.

### 2.3 SCT Exerts Its Protective Effects by Enhancing Mitophagy

**FIGURE 3:**
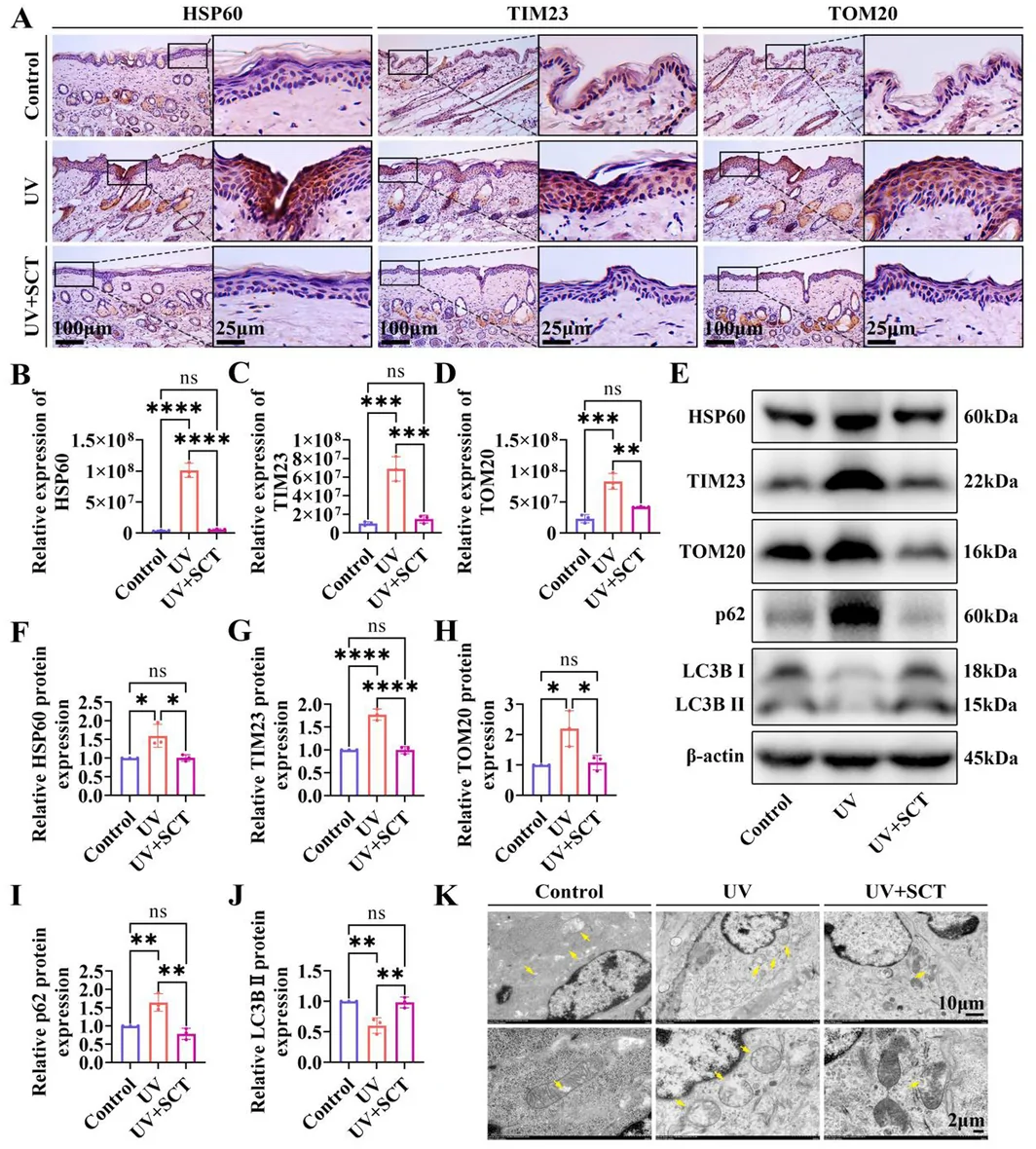
SCT enhances mitophagy in KM mice. (A–D) Immunohistochemical staining and statistics of relative levels of HSP60, TIM23 and TOM20 in the epidermis, *n* = 3 (scale bar, 100 μm). (E–J) Western blotting was used to measure HSP60, TIM23 and TOM20 expression in skin tissues, *n* = 3. (K) TEM was performed to observe the ultrastructure of the skin tissues of mice, and the formation of mitochondria (yellow arrows) was observed (scale bar, 500 nm). *p < 0.05, **p < 0.01, ***p < 0.001, and ****p < 0.0001.

The potential mechanism by which SCT exerts its protective effects was further investigated, with a focus on mitochondrial quality control, a key process in maintaining cellular homeostasis. Immunohistochemical and western blot analyses revealed that UV irradiation significantly elevated the levels of mitochondrial structural proteins (TOM20, TIM23, and HSP60) and the autophagy adaptor p62, while reducing LC3B‐II levels in mouse skin tissues. The concurrent accumulation of p62 and mitochondrial proteins, together with decreased LC3B‐II, suggests impaired autophagic clearance of damaged mitochondria. Notably, SCT administration effectively reversed all of these alterations, indicating that SCT promotes mitophagy activation (Figure 3A–J).

We next asked whether SCT engages the PINK1/Parkin pathway, a canonical mitophagy regulatory axis. Western blotting showed that UV irradiation significantly reduced PINK1 and Parkin expression in mouse skin, whereas SCT treatment upregulated their levels (Figure S4A–C). These findings suggest that SCT‐triggered mitophagy in vivo is associated, at least in part, with PINK1/Parkin upregulation.

Transmission electron microscopy (TEM) further demonstrated that mitochondria in skin cells of the control group exhibited normal morphology with intact and well‐organized cristae. In contrast, the UV group displayed severe ultrastructural damage, including mitochondrial matrix swelling, cristae fragmentation, and vacuolation. These abnormalities were markedly alleviated by SCT administration (Figure 3K), further supporting the notion that SCT facilitates the clearance of damaged mitochondria via mitophagy.

**FIGURE 4:**
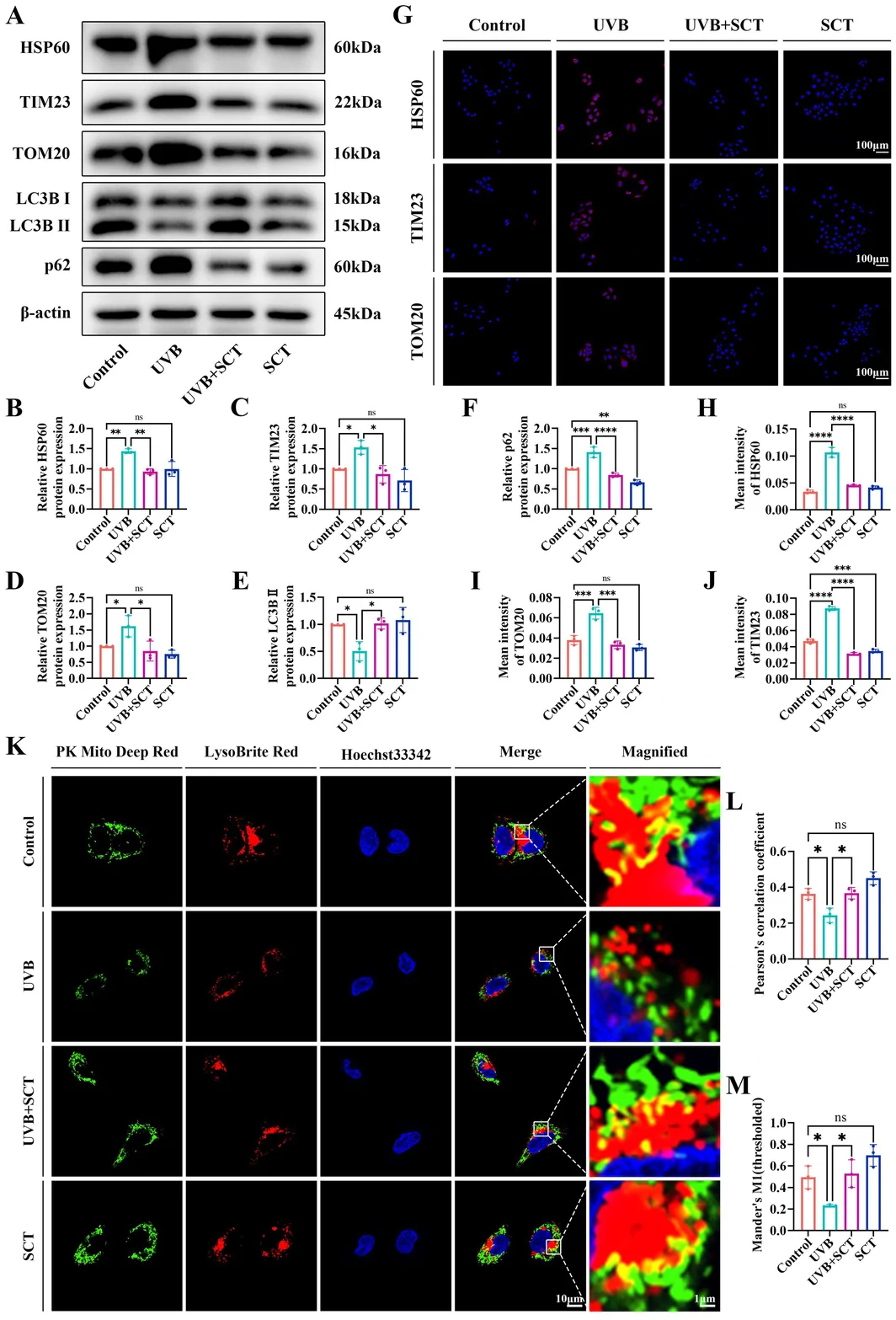
SCT enhances mitophagy during the recovery of UVB‐injured HaCaT cells. (A–F) Western blotting was used to measure HSP60, TIM23, TOM20, p62 and LC3B‐II expression in HaCaT cells, *n* = 3. (G–J) Immunofluorescence staining and statistics of relative levels of HSP60, TIM23 and TOM20 in HaCaT cells, *n* = 3 (scale bar, 100 μm). (K) Confocal co‐localization micrographs stained with PK Mito Deep Red (mitochondria, green), LysoBrite Red (lysosome, red) and Hoechst 33342 (nucleus, blue); magnified insets visualize overlapping fluorescence signals (scale bar, 10 μm, magnified inset scale bar, 1 μm). Pearson's correlation coefficient (R) is annotated for each group. (L) Quantitative statistical analysis of Pearson's correlation coefficient for mitochondrial–lysosome co‐localization. (M) Quantification of Manders' thresholded overlap coefficient M1 (mitochondrial signal overlapping lysosomal signal). *p < 0.05, **p < 0.01, ***p < 0.001, and ****p < 0.0001.

Consistent with the in vivo observations, UVB‐exposed HaCaT cells showed increased p62, TOM20, TIM23, and HSP60 along with decreased LC3B‐II; these changes were reversed by SCT treatment (Figure 4A–F). Similarly, PINK1 and Parkin were downregulated after UVB exposure and restored by SCT (Figure S5A–C), suggesting that SCT‐promoted mitophagy in vitro is also associated with elevated PINK1/Parkin levels. Immunofluorescence staining corroborated these findings, showing increased TOM20, TIM23, and HSP60 signals after UVB exposure, which were significantly reduced by SCT treatment (Figure 4G–J).

To obtain direct evidence of mitophagy activation, mitochondrial–lysosomal colocalization was assessed using the probes PK Mito Deep Red and LysoBrite. Confocal imaging showed that relative to the UVB group, UVB + SCT treatment notably increased the overlapping fluorescent signal between mitochondria and lysosomes, as clearly demonstrated in high‐magnification images (Figure 4K, insets). Quantitative analysis was performed on multiple randomly selected cells from three independent experiments. Both Pearson's correlation coefficient and Manders' overlap coefficient M1—where M1 represents the fraction of mitochondrial (PK Mito Deep Red) fluorescence that overlaps with lysosomal (LysoBrite) signal—were significantly higher in the UVB + SCT group than in the UVB group (Figure 4L,M). In addition, conventional MitoTracker/LysoTracker co‐staining was performed, and Pearson's coefficients further confirmed enhanced mitochondrial–lysosomal colocalization after SCT intervention under UVB stimulation (Figure S5D–H).

To ascertain whether SCT ameliorates photoaging via restoring mitophagy, CCCP (a mitochondrial uncoupler that triggers mitophagy) and Mdivi‐1 (a Drp1 inhibitor commonly used to block mitophagy) were employed. CCK‐8 assays were conducted to determine optimal treatment concentrations. 1 μM CCCP and 7 μM Mdivi‐1 were identified as optimal concentrations that maintained cell viability (Figure S6A,B). Using Lamin B1 as a readout for cellular senescence, western blot analysis revealed that compared with the UVB + SCT group, CCCP co‐treatment further enhanced Lamin B1 expression, whereas Mdivi‐1 significantly attenuated the SCT‐induced upregulation of Lamin B1 (Figure S6C,D). Notably, CCCP treatment alone recapitulated the protective effect of SCT against UVB‐induced senescence, further supporting the model that SCT exerts its anti‐photoaging effects largely via mitophagy restoration.

Collectively, these results demonstrate that SCT restores UV/UVB‐suppressed mitophagy both in vivo and in vitro, and that this restoration contributes substantially to its anti‐photoaging effects.

### 2.4 Mitophagy Inhibits the cGAS‐STING Signaling Pathway

**FIGURE 5:**
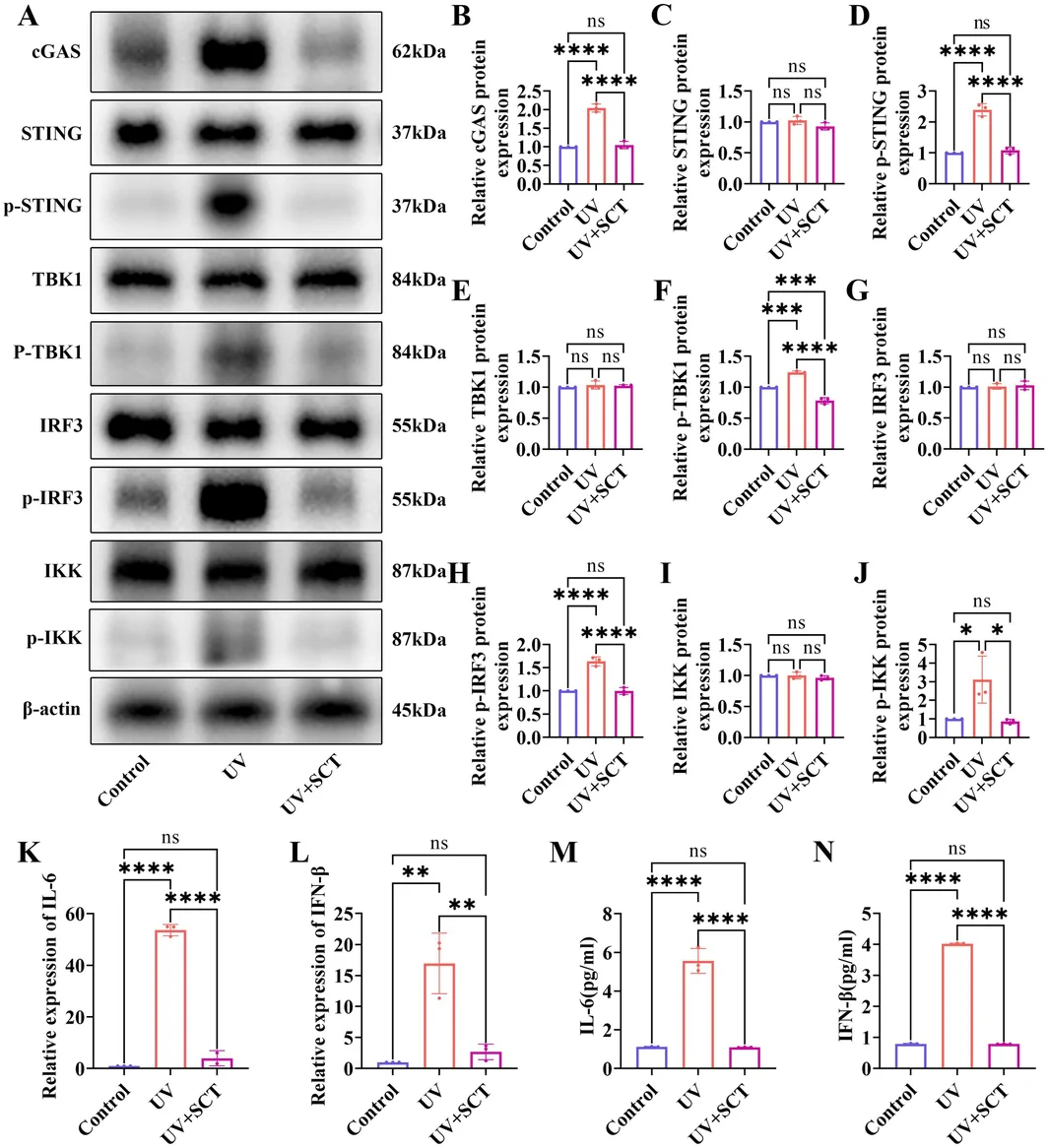
SCT inhibits the cGAS‐STING signaling pathway in KM mice. (A–J) Western blotting was used to measure cGAS, p‐STING, STING, p‐TBK1, TBK1, p‐IRF3, IRF3, p‐IKK and IKK expression in skin tissues, *n* = 3. (K, L) RT‐qPCR was performed to measure IL‐6 and IFN‐β expression in the skin tissues of mice, *n* = 3. (M, N) Elisa was performed to measure the concentrations of IL‐6 and IFN‐β in mouse serum, *n* = 3. *p < 0.05, **p < 0.01, ***p < 0.001, and ****p < 0.0001.

Based on the premise that mitochondrial damage can lead to the release of mtDNA into the cytoplasm, thereby activating the cGAS‐STING innate immune signaling pathway, we hypothesized that SCT might inhibit the overactivation of this pathway by enhancing mitophagy to clear damaged mitochondria and leaked mtDNA. To test this hypothesis, we first evaluated whether the cGAS‐STING pathway is activated in the photoaging model and whether SCT influences its activity. Western blot analysis revealed that, compared with the control group, the expression of cGAS and the phosphorylated forms of STING (p‐STING), TBK1 (p‐TBK1), IRF3 (p‐IRF3), and IKK (p‐IKK) was significantly increased in UV‐irradiated mouse skin tissues, indicating activation of this pathway during photoaging. Importantly, SCT treatment effectively reversed these UV‐induced changes, significantly downregulating the expression of these key pathway components (Figure 5A–J). Analysis of inflammatory factors by RT‐qPCR showed that UV irradiation markedly elevated the mRNA levels of IL‐6 and IFN‐β in mouse skin, while SCT treatment effectively suppressed the expression of both cytokines (Figure 5K,L). ELISA results of serum samples further confirmed the anti‐inflammatory effect of SCT at the systemic level (Figure 5M,N).

To visualize cytosolic dsDNA accumulation, we performed fluorescence co‐staining using PicoGreen (a dsDNA probe) and MitoDeepRed (a mitochondrial marker) in HaCaT cells. Confocal imaging showed that UVB irradiation significantly increased cytosolic dsDNA signals (defined as PicoGreen^+^ signals outside MitoDeepRed‐labeled mitochondria), while SCT co‐treatment markedly reversed this accumulation (Figure S7A–C). These findings are consistent with reduced mtDNA leakage upon SCT treatment, although PicoGreen does not independently establish the mitochondrial origin of the detected dsDNA.

**FIGURE 6:**
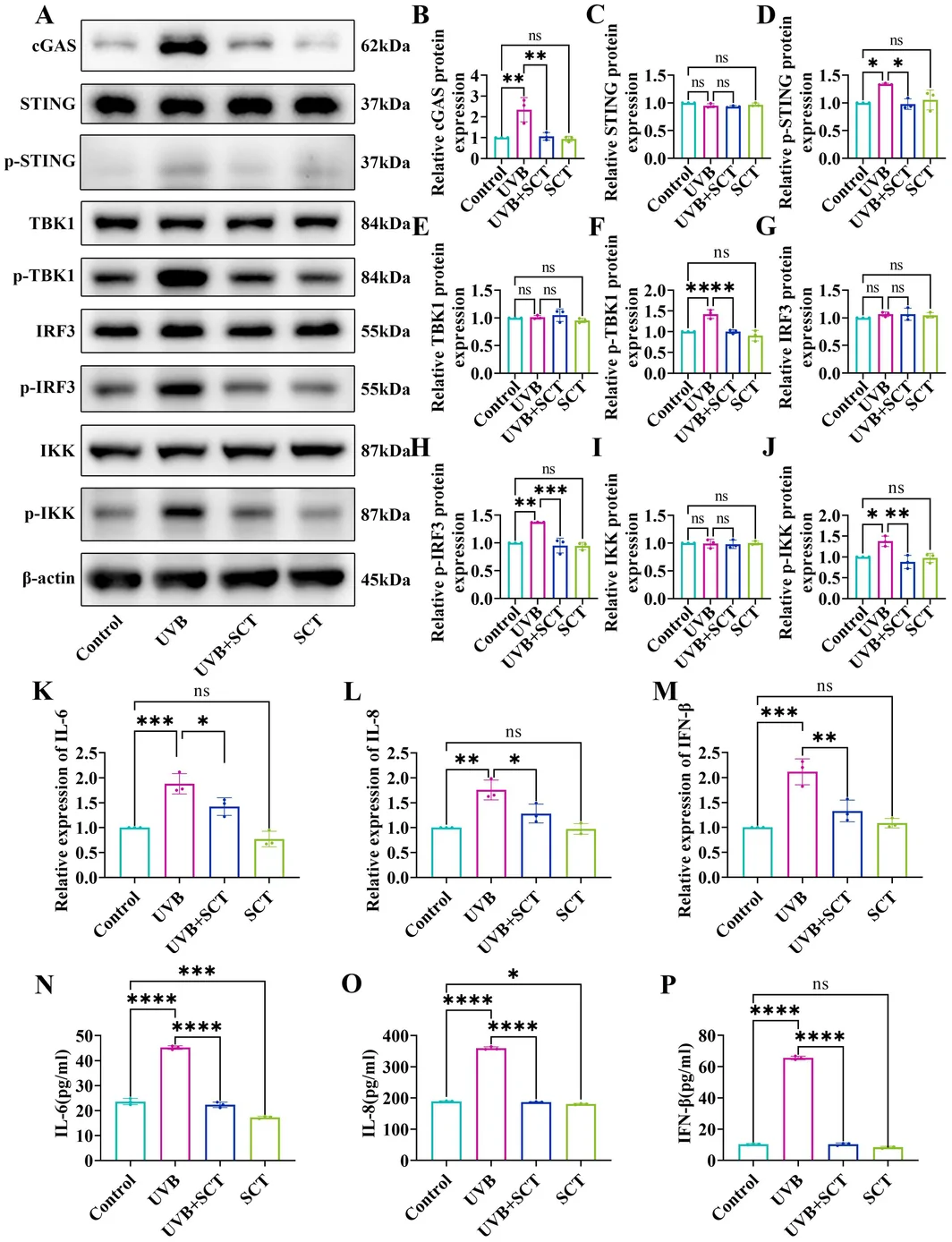
SCT inhibits the cGAS‐STING signaling pathway and promotes the recovery of UVB‐injured HaCaT cells. (A–J) Western blotting was used to measure cGAS, p‐STING, STING, p‐TBK1, TBK1, p‐IRF3, IRF3, p‐IKK, and IKK expression in HaCaT cells, *n* = 3. (K–M) RT‐qPCR was performed to measure IL‐6, IL‐8, and IFN‐β expression in HaCaT cells, *n* = 3. (N–P) Elisa was performed to measure the concentrations of IL‐6, IL‐8, and IFN‐β in HaCaT cell culture supernatants, *n* = 3. *p < 0.05, **p < 0.01, ***p < 0.001, and ****p < 0.0001.

Similarly, in the UVB‐irradiated HaCaT cell model, Western blot analysis demonstrated a significant upregulation in the protein expression of key components of the cGAS‐STING pathway. Specifically, the levels of cGAS, the phosphorylated forms of STING (p‐STING), TBK1 (p‐TBK1), IRF3 (p‐IRF3), and IKK (p‐IKK) were markedly increased compared to the non‐irradiated control group. This confirmed the activation of the innate immune signaling pathway in keratinocytes following UVB irradiation. Crucially, intervention with SCT effectively reversed this activation, leading to a significant downregulation of these proteins (Figure 6A–J). Consistent with the protein data, RT‐qPCR analysis revealed that UVB irradiation robustly stimulated the mRNA expression of the downstream inflammatory cytokines IL‐6, IL‐8, and IFN‐β. Pre‐treatment with SCT, however, potently suppressed this effect, markedly reducing the mRNA levels of these pro‐inflammatory factors (Figure 6K–M). Furthermore, to directly assess the secretory inflammatory response, the concentrations of IL‐6, IL‐8, and IFN‐β were measured in the cell culture supernatant. ELISA results indicated that UVB irradiation induced a substantial release of these cytokines. Again, SCT treatment significantly attenuated their secretion (Figure 6N–P), providing direct evidence of its anti‐inflammatory action at the cellular level. These results indicate that SCT effectively suppresses cGAS‐STING pathway activation and downstream inflammatory responses in vitro.

To determine whether SCT acts through the regulation of the cGAS‐STING pathway, we employed the STING agonist MSA‐2 and inhibitor H151 for intervention. CCK‐8 assays determined that 30 μM MSA‐2 and 7 μM H151 were suitable concentrations for subsequent experiments (Figure S8A,B). Western blot analysis showed that the STING agonist MSA‐2 attenuated the ameliorative effect of SCT on cellular senescence, whereas the inhibitor H151 further enhanced the protective effect of SCT (Figure S8C,D), suggesting that suppression of the cGAS‐STING pathway contributes to SCT‐mediated anti‐photoaging effects.

To elucidate the regulatory role of mitophagy in the cGAS‐STING‐mediated inflammatory response, functional experiments using pharmacological modulators were conducted. Western blot analysis revealed that the mitophagy agonist CCCP significantly downregulated the protein levels of key cGAS‐STING pathway components, including cGAS, p‐STING, p‐TBK1, p‐IRF3, and p‐IKK, in UVB‐irradiated HaCaT cells (Figure S9A–J). In contrast, the mitophagy inhibitor Mdivi‐1 markedly elevated these protein expression levels under the same conditions. Consistent with the protein data, ELISA measurements of the cell culture supernatant revealed that CCCP treatment potently decreased the UVB‐induced secretion of the pro‐inflammatory cytokines IL‐6, IL‐8, and IFN‐β, whereas Mdivi‐1 treatment markedly elevated their secretion (Figure S9K–M). Further experiments using the STING agonist MSA‐2 and inhibitor H151 under UVB irradiation showed that, while MSA‐2 further enhanced the UVB‐induced secretion of IL‐6, IL‐8, and IFN‐β, H151 effectively reduced their levels, as measured by ELISA analysis of the cell culture supernatant (Figure S9N–P). Collectively, these results indicate that enhancing mitophagy inhibits the cGAS‐STING pathway and its downstream inflammatory response, whereas inhibiting mitophagy exerts opposing effects on pathway activity and the inflammatory response.

Collectively, these results indicate that enhancing mitophagy suppresses the cGAS‐STING‐mediated inflammatory response, likely through clearance of damaged mitochondria and leaked mtDNA.

### 2.5 SCT Ameliorates Photoaging via the Mitophagy–cGAS‐STING Axis

To investigate whether SCT ameliorates photoaging through the mitophagy–cGAS‐STING axis, we conducted rescue experiments at the cellular level using a dual‐target intervention strategy with the mitophagy inhibitor Mdivi‐1 and the STING‐specific inhibitor H151.

Western blot analysis demonstrated that, compared to the UVB irradiation group alone, UVB + SCT treatment significantly enhanced the expression of mitophagy‐related proteins and suppressed the phosphorylation of key proteins in the cGAS‐STING pathway. When the STING inhibitor H151 was added following UVB + SCT treatment, mitophagy activity was not significantly affected, as evidenced by expression levels of mitophagy‐related proteins that remained similar to those in the UVB + SCT group (Figure S10A–F). However, inhibition of the cGAS‐STING signaling pathway was further enhanced, as evidenced by a greater reduction in the phosphorylation levels of p‐STING, p‐TBK1, p‐IRF3, and p‐IKK compared to the UVB + SCT group (Figure S10G–P). This supports the view that suppression of this signaling axis contributes substantially to SCT‐dependent protective effects.

In contrast, when the mitophagy inhibitor Mdivi‐1 was introduced, SCT‐induced mitophagy was completely blocked. This was reflected by a marked increase in the levels of mitochondrial structural proteins HSP60, TIM23, and TOM20, a clear accumulation of the autophagy adaptor protein p62, and a decrease in the expression of the autophagosome marker LC3B‐II (Figure S10A–F). Concurrently, the inhibitory effect of SCT on the cGAS‐STING pathway was substantially attenuated, with elevated phosphorylation levels of p‐STING, p‐TBK1, p‐IRF3, and p‐IKK (Figure S10G–P). These findings indicate that SCT‐mediated suppression of the cGAS‐STING pathway is dependent on the functional integrity of mitophagy.

In the Mdivi‐1 and H151 co‐treated group, although mitophagy remained suppressed (Figure S10A–F), the addition of H151 effectively reversed the activating effect of Mdivi‐1 and reduced the phosphorylation levels of these proteins to levels similar to those in the UVB + SCT group (Figure S10G–P). This crucial result demonstrates that Mdivi‐1 counteracts SCT's effects by inhibiting mitophagy, thereby activating the cGAS‐STING pathway; importantly, H151 can directly suppress STING to bypass the mitophagy blockade and restore the protective effect of SCT. Thus, this pharmacological epistasis observation is consistent with a hierarchical relationship in which mitophagy acts upstream of cGAS‐STING signaling.

Consistent with these protein‐level changes, the expression of inflammatory mediators followed a similar trend. ELISA results showed that Mdivi‐1 reversed the inhibitory effects of UVB + SCT on the transcription and secretion of IL‐6, IL‐8, and IFN‐β, whereas the addition of H151 reversed this Mdivi‐1‐induced inflammatory rebound, bringing cytokine levels back to those observed in the UVB + SCT treatment group (Figure S11A–C). This further confirms that STING is the principal driver of the inflammatory response in this model.

At the phenotypic level, the mitophagy inhibitor impaired the ability of UVB + SCT to ameliorate UV‐induced photoaging characteristics. However, additional STING inhibition not only reversed this effect but also restored the full protective capacity of SCT against cellular photoaging (Figure S11D,E), demonstrating that the deleterious effects caused by mitophagy blockade are predominantly mediated through STING activation.

In conclusion, these cellular rescue assays support a hierarchical model whereby SCT alleviates keratinocyte photoaging primarily through enhancing mitophagy, which in turn dampens cGAS‐STING‐driven inflammation and senescence progression. Importantly, our data further demonstrate that STING represents a non‐redundant and obligate downstream hub in this pathway, as its direct inhibition is sufficient to bypass the requirement for upstream mitophagy and still confer protection against photoaging.

### 2.6 SCT Ameliorates Photoaging via the Mitophagy–cGAS‐STING Axis In Vivo

To validate the hierarchical mitophagy–cGAS–STING cascade identified in our cellular rescue assays, we employed a dual‐pharmacological epistasis design using the mitophagy inhibitor Mdivi‐1 and the STING inhibitor H151 in both in vitro and in vivo models. This approach also helped minimize the confounding effects associated with pharmacological tools.

We first assessed mitophagy regulatory proteins in skin tissues. Western blot analysis showed that the SCT‐induced upregulation of PINK1 and Parkin was markedly abrogated by Mdivi‐1 co‐administration, accompanied by accumulation of the mitochondrial matrix chaperone HSP60 (Figure S12A–D). These data confirm that Mdivi‐1 effectively blunts SCT‐driven mitophagy in vivo. Mitophagy marker expression in the UV + SCT + H151 group remained comparable to the UV + SCT group, consistent with H151 targeting STING downstream of mitophagy regulation. In the Mdivi‐1 plus H151 co‐treatment group, mitophagy remained suppressed, as reflected by low PINK1/Parkin levels and sustained HSP60 elevation (Figure S12A–D). Collectively, these results confirm that SCT‐augmented mitophagy is blocked by Mdivi‐1, whereas downstream STING inhibition does not feed back on this process.

Having established the effects of Mdivi‐1 and H151 on mitophagy, we next examined cGAS–STING pathway activation. Mdivi‐1 largely reversed the suppressive effect of SCT on this pathway, as evidenced by elevated cGAS protein and enhanced phosphorylation of STING (Figure S12E–H). In contrast, H151 further enhanced the inhibitory effect of SCT, as shown by greater reductions in these phosphorylated proteins compared with the UV + SCT group. Notably, in the Mdivi‐1 plus H151 co‐treatment group—where mitophagy remained suppressed—H151 effectively reversed the Mdivi‐1‐induced elevation of phosphorylated proteins, bringing their levels back to those seen in the UV + SCT group (Figure S12E–H). These findings support a hierarchical model in which SCT suppresses cGAS–STING signaling through mitophagy enhancement, whereas direct STING inhibition can bypass upstream dysfunction to shut down this pro‐inflammatory axis.

We next assessed downstream inflammatory outputs by measuring serum cytokines via ELISA. Consistent with the molecular changes, Mdivi‐1 significantly reversed the SCT‐mediated reduction in serum IL‐6 and IFN‐β levels. Notably, H151 co‐administration effectively counteracted this Mdivi‐1‐induced elevation, restoring cytokine levels to those comparable with the UV + SCT group (Figure S12I,J). These results support the notion that SCT mitigates systemic inflammation in vivo through the mitophagy–cGAS–STING axis.

Lastly, we assessed senescence markers in skin tissue. As anticipated, mitophagy inhibition by Mdivi‐1 compromised SCT‐dependent restoration of Lamin B1, leading to significantly reduced Lamin B1 levels (Figure S12K,L). Strikingly, additional STING inhibition with H151 reversed this phenotype and restored Lamin B1 expression to levels similar to the UV + SCT group, indicating that the anti‐senescence effect of SCT requires intact signaling through this axis.

Together, these in vivo findings demonstrate that SCT protects against photoaging by promoting mitophagy, which in turn constrains cGAS–STING activation, attenuates systemic inflammation, and alleviates cellular senescence. These experiments not only corroborate our cell‐based rescue assays but also establish the mitophagy–cGAS–STING axis as a functionally relevant and therapeutically targetable node in cutaneous photoaging.

## 3 Discussion

### 3.1 SCT Ameliorates Both Epidermal and Dermal Photoaging Phenotypes

Skin photoaging is an extrinsic skin disorder characterized clinically by wrinkle formation, pigmentation, loss of elasticity, and an increased risk of carcinogenesis (Gargano et al. 2026; Jin et al. 2025). The secretome, a cell‐free therapeutic agent derived from mesenchymal stem cells, demonstrates remarkable immunomodulatory and tissue‐repair capabilities due to its rich content of bioactive factors, including cytokines, growth factors, and extracellular vesicles. Studies have shown that hUC‐MSC‐derived SCT upregulates key growth factors such as VEGF‐A and PDGF‐AA, significantly promoting embryonic development and endometrial cell proliferation (Teerapong et al. 2025).

The present study confirms that SCT significantly ameliorates ultraviolet‐induced skin photoaging. Our findings confirm that SCT markedly ameliorates UV‐induced skin photoaging. In animal models, SCT reversed UV‐driven epidermal thickening, loss of elasticity, and barrier dysfunction. At the cellular level, SCT reduced the percentage of SA‐β‐gal‐positive HaCaT cells and reversed UVB‐mediated Lamin B1 downregulation, demonstrating direct anti‐senescence activity. SCT also suppressed UV‐induced upregulation of the cell‐cycle arrest markers p21 and p16 in the epidermis, reinforcing this conclusion. We have characterized these epidermal mechanisms, with particular focus on the mitochondrial inflammaging axis in keratinocytes.

Beyond epidermal protection, SCT also preserved dermal collagen integrity by restoring COL1 and COL3 abundance while suppressing MMP1/3/9 upregulation, indicating that SCT counteracts the two cardinal pathological features of photoaging: epidermal barrier disruption and dermal matrix degradation. The matrix‐protective phenotype we observed is consistent with previous reports that hUC‐MSC‐derived exosomes restore skin barrier function in atopic dermatitis models (Shao et al. 2025). However, the molecular pathways through which SCT influences dermal remodeling remain incompletely defined. Future work will be essential to dissect these mechanisms, including the roles of paracrine signaling between epidermal and dermal compartments, the involvement of specific growth factors and cytokines in mediating fibroblast function, and the broader regulatory networks that coordinate matrix homeostasis.

### 3.2 SCT Enhances Mitophagy to Suppress the cGAS‐STING Pathway in Keratinocytes

Mechanistically, our findings identify that SCT ameliorates skin photoaging via the mitophagy–cGAS‐STING axis. SCT enhanced mitophagy both in vivo and in vitro, reversing UV‐driven accumulation of mitochondrial damage markers (p62 and TOM20) and restoring mitochondrial cristae architecture, as visualized by transmission electron microscopy. Increased mitochondrial–lysosomal colocalization further confirmed recovery of autophagic flux. Furthermore, we implicate the canonical PINK1/Parkin mitophagy cascade in SCT‐mediated mitochondrial clearance.

SCT also markedly suppressed cGAS–STING pathway overactivation, as reflected by reduced phosphorylation of STING, TBK1, and IRF3 in skin tissue, decreased serum IL‐6 and IFN‐β, and lower IL‐8 secretion from cultured keratinocytes. Mechanistically, SCT‐restored mitophagy limits cytosolic mtDNA leakage, providing a plausible link between mitochondrial quality control and cGAS–STING inhibition. Imaging evidence showing that SCT reduced UVB‐triggered cytosolic dsDNA accumulation supports this interpretation.

The regulatory cascade we describe here differs substantially from tissue‐repair signaling axes reported in other disease models, such as VEGF/HGF‐mediated angiogenesis (Lu et al. 2013), highlighting its distinct therapeutic relevance to UV‐induced skin damage. Moreover, although previous studies have shown that mitophagy curbs mtDNA leakage and suppresses cGAS–STING in cardiac hypertrophy and neurodegenerative diseases (Zhou et al. 2025; Akhter et al. 2025), the present work provides direct cutaneous in vivo evidence for this cascade, extending those mechanistic insights into the skin aging context.

Whereas earlier studies have emphasized the anti‐inflammatory actions of hUC‐MSCs primarily through the STAT3 pathway (Shao et al. 2025), our work identifies the mitophagy**–**cGAS‐STING axis as a critical effector mechanism in photoaging and demonstrates that the protective effect is mediated paracrinally by secretory components rather than by cell transplantation. This distinction offers new perspectives for the development of cell‐free therapies.

### 3.3 Rationale for the UV Irradiation Protocol and Animal Model

We consider it important to contextualize the UV regimen used in this study. Our UVA:UVB intensity ratio (~6.7:1) approximates that of terrestrial sunlight, in which UVA predominates and UVB declines steeply with decreasing wavelength because of stratospheric ozone absorption (Diffey 1991). Our progressive exposure schedule (10–30 min/day) yielded total cumulative doses of 63 J/cm^2^ (UVA) and 9.45 J/cm^2^ (UVB), with daily averages of ~1.6 J/cm^2^ and 0.24 J/cm^2^, respectively. This regimen mimics chronic sub‐erythemal solar exposure and allows skin adaptation, reflecting long‐term repeated exposure rather than acute insult. Our UVB dose rate (0.15 mW/cm^2^) is comparable to that used in established photodamage models (Sharma et al. 2011). No acute phototoxic reactions were observed over the 40‐day protocol, confirming that the regimen effectively minimized acute damage while reliably eliciting chronic photoaging phenotypes. The validity of this approach is further supported by our previous work (Lin et al. 2023), in which the same protocol produced characteristic photoaging phenotypes closely paralleling human photoaged skin.

### 3.4 Conclusion

In conclusion, this study provides compelling preclinical evidence that SCT is a potent therapeutic agent for ameliorating skin photoaging. We have identified a novel mechanism of action involving restoration of mitochondrial homeostasis through enhanced mitophagy and subsequent suppression of cGAS–STING‐mediated inflammation. These findings establish the mitophagy–cGAS–STING axis as a promising therapeutic target for anti‐photoaging treatment and highlight the translational potential of cell‐free MSC secretome in regenerative dermatology, with broader implications for other mitochondrial‐dependent inflammatory aging disorders. While our experiments were conducted in murine models and in vitro cell cultures—systems that cannot fully recapitulate the complexity of human skin—these findings warrant further clinical validation to translate them into effective therapies for patients with photoaged skin.

## 4 Materials and Methods

### 4.1 Preparation of hUC‐MSC‐Derived SCT

Human umbilical cord‐derived mesenchymal stem cells (hUC‐MSCs) were isolated and cultured following our previously standardized protocol (Zhang, Wang, et al. 2025). Briefly, hUC tissue was obtained from a single healthy full‐term donor with signed informed consent and institutional ethical approval. Wharton's jelly was separated, cut into 3–5 mm^3^ fragments, and seeded in serum‐free hUC‐MSC complete medium (NC0106 + NC0106.S, Yocon, China) supplemented with 1% penicillin/streptomycin (ScienCell, USA) at 37°C in a 5% CO_2_ atmosphere. Cells were passaged upon reaching 95% confluence and seeded at a density of 8000 cells/cm^2^ in 10 cm flasks. Only passage 5 (P5) hUC‐MSCs were used for secretome harvest, given their stable spindle morphology and superior proliferative capacity compared to later passages (Zhang, Wang, et al. 2025). For secretome collection, P5 cells were cultured for an additional 72 h in serum‐free medium after reaching full confluence. The conditioned medium was harvested and sequentially filtered through a 40‐μm cell strainer (Biologix, China) to remove cell debris, followed by 0.22‐μm sterile filtration (Millipore, USA) to ensure sterility. The total protein concentration of the stock SCT was measured as 2.548 ± 0.103 mg/mL (*n* = 10) (Zhang, Wang, et al. 2025). All SCT used in this study was derived from a single donor batch to minimize batch‐to‐batch variability; no pooling of multiple donors was performed. The clarified SCT was dispensed into single‐use aliquots (15‐mL centrifuge tubes) and stored at −80°C; repeated freeze–thaw cycles were strictly avoided before use. For in vivo topical application, undiluted stock SCT (2.548 mg/mL protein) was applied at 200 μL per mouse daily (equivalent to approximately 509.6 μg protein per mouse per day). For in vitro experiments, SCT was diluted in serum‐free DMEM to final concentrations ranging from 0% to 70% (v/v); 50% SCT (1.274 mg/mL protein) was selected as the optimal working concentration based on CCK‐8 viability assays. Vehicle controls received equal volumes of PBS.

### 4.2 Mouse Photoaging Model Establishment and Treatments

Five‐month‐old female Kunming (KM) mice were obtained from the Experimental Animal Center of Southern Medical University (Guangzhou, China). After 7 days of acclimatization, the mice were randomly divided into four groups: control group (Control), SCT‐only group (SCT, no UV irradiation), ultraviolet irradiation model group (UV), and ultraviolet irradiation combined with SCT treatment group (UV + SCT).

The SCT‐only group received identical dorsal depilation procedures but no UV exposure; 200 μL SCT solution was topically applied daily for 30 days, matching the treatment schedule of the UV + SCT group. Control and UV groups received equal‐volume PBS vehicle.

Twenty‐four hours before initiating UV exposure, the dorsal hair was removed using a mild depilatory cream. Throughout the 40‐day irradiation period, depilation was repeated every 5–7 days according to the hair regrowth cycle. To avoid potential interference of depilation with UV effects, irradiation was consistently performed 12 h after each depilation. The irradiation area was standardized to a defined dorsal skin region (2.5 × 1.5 cm).

Mice were immobilized using a custom‐made restraint device. Non‐irradiated areas were covered with aluminum foil for complete UV protection, and animals were placed in a self‐constructed UV irradiation chamber. The chronic photoaging model was established over 40 days using UVA (340 nm, 40 W) and UVB (313 nm, 40 W) irradiation at intensities of 1 mW/cm^2^ and 0.15 mW/cm^2^, respectively. UV intensities were verified using a calibrated radiometer at the beginning of the experiment and weekly thereafter to ensure consistent output throughout the protocol. The UVA:UVB intensity ratio (≈6.7:1) is consistent with the spectral characteristics of terrestrial sunlight, in which UVA predominates and UVB decreases sharply with decreasing wavelength due to stratospheric ozone absorption (Diffey 1991). Furthermore, the selected wavelengths—340 nm for UVA and 313 nm for UVB—represent physiologically relevant UV components that reach the Earth's surface, as UVC is completely absorbed by the ozone layer and approximately 90% of UVB is absorbed atmospherically (Bahamondes Lorca et al. 2022). Unlike conventional UVB lamps that emit non‐physiological UVC wavelengths (270–280 nm) absent in natural sunlight, our 313 nm source avoids such contamination, further supporting the environmental relevance of the model. The total cumulative doses over the 40‐day protocol were 63 J/cm^2^ for UVA and 9.45 J/cm^2^ for UVB. The progressive exposure regimen (10 min/day initially, gradually increasing to 30 min/day) was adopted to minimize inter‐individual variability and avoid acute skin injury. No acute phototoxic reactions (e.g., ulceration, severe erythema, or desquamation) were observed in any UV‐exposed animals throughout the 40‐day protocol, confirming that this regimen effectively minimized acute injury while reliably inducing chronic photoaging phenotypes. The combined use of UVA and UVB takes advantage of their synergistic effects in inducing extracellular matrix degradation and epidermal hyperplasia, allowing for reliable photoaging phenotype establishment within a 40‐day period. This irradiation protocol was systematically validated in our previous work (Lin et al. 2023), where it induced characteristic chronic photoaging phenotypes without acute phototoxicity, paralleling human photoaged skin.

After 10 days of adaptive irradiation, topical treatment was initiated. The UV + SCT group received 200 μL of SCT solution, while the Control and UV groups received an equal volume of PBS. Treatments were administered 30 min after daily irradiation and continued for 30 days.

Upon completion of the 40‐day irradiation protocol, mice were anesthetized via intraperitoneal injection of 1% sodium pentobarbital. Macroscopic images were first captured (Trichoscan HD, Germany), followed by measurements of transepidermal water loss (TEWL) (AF200, Biox system, UK), skin elasticity, and epidermal hydration (DUB‐MSTC, TPM, Germany). Serum samples and irradiated skin tissues were then collected for subsequent experimental analyses.

All animal experiments were approved by the Ethics Committee of Southern Medical University (experimental protocol code: SMUL2023054, 14 April 2023).

### 4.3 In‐Vivo Pharmacological Rescue Experiments

For in vivo mitophagy–cGAS‐STING axis rescue assays, KM mice subjected to 40‐day UV photoaging were allocated to four additional groups: UV + SCT, UV + SCT + Mdivi‐1, UV + SCT + H151, UV + SCT + Mdivi‐1 + H151. Mdivi‐1 (intraperitoneal injection, 7 mg/kg every other day) and H151 (intraperitoneal injection, 5 mg/kg every other day) were administered according to pre‐optimized protocols, with the first injection given on Day 11, i.e., the first day of SCT topical treatment. Corresponding vehicle solvent was given to control groups. SCT topical treatment was maintained as described above. At experimental endpoint, skin tissues and serum were collected for Western blot and ELISA.

### 4.4 Hematoxylin and Eosin (H&E) Staining

Following collection of dorsal skin lesions in mice, tissues were rinsed using phosphate‐buffered saline (PBS), fixed with 4% paraformaldehyde (PFA), dehydrated through an ethanol series, paraffin‐embedded, sectioned at 5‐μm thickness, hematoxylin and eosin (H&E) stained, and subjected to microscopic evaluation for histological analysis.

### 4.5 Masson's Trichrome Staining

Paraffin‐embedded dorsal mouse skin sections were de‐paraffinized and rehydrated. Masson's trichrome staining was performed following the manufacturer's protocol. Sections were mounted and imaged under light microscopy. Dermal collagen fiber integrity and relative collagen content were evaluated; quantitative analysis of collagen fraction was performed using ImageJ software on randomly selected fields.

### 4.6 Immunohistochemistry Staining

The immunohistochemical staining procedure involved incubating the samples overnight at a temperature of 4°C with primary antibodies including Lamin B1 (1:300, Abcam, ab133741, UK), p21 (1:250, Abmart, TD6423F, China), p16 (1:250, Abmart, PC23797S, China), COL1 (1:1000, Proteintech‐cn, 14695‐1‐AP, USA), COL3 (1:100, Abmart, T510299F, China), MMP1 (1:100, Abmart, PA1747F, China), MMP3 (1:100, Abmart, T55188F, China), MMP9 (1:100, MCE, HY‐P80756, USA), HSP60 (1:250, Proteintech‐cn, 15282‐1‐AP, USA), TIM23 (1:250, Proteintech‐cn, 11123‐1‐AP, USA), TOM20 (1:1000, Proteintech‐cn, 11802‐1‐AP, USA). Following this, the samples were rinsed with PBS and subsequently incubated with either biotinylated goat anti‐rabbit secondary antibody (ZSGB‐BIO, PV‐6001, China) for 1 h. The tissue slices were then subjected to immunohistochemical staining using 3,3‐diaminobenzidine (DAB) (1:20, ZSGB‐BIO, ZLI‐9017, China), followed by hematoxylin staining. Subsequently, the slices were dehydrated using a gradient ethanol series, treated with xylene, and sealed with resin. The resulting images were observed and captured using a microscope.

### 4.7 TEM

Fresh skin tissues (1 mm^3^) were fixed in 2.5% glutaraldehyde. The tissues were dehydrated in gradient ethanol solutions, embedded, and sectioned. The sections were stained with uranyl acetate and lead citrate. TEM was used for visualization.

### 4.8 Western Blotting Analysis

Samples were lysed on ice for 30 min using RIPA lysis buffer (Beyotime, P0013, China) followed by centrifugation at 4°C and 12,800 rpm for 15 min. The supernatant was collected, and the protein concentration was determined using the bicinchoninic acid (BCA) assay (Beyotime, P0010, China). A volume of 5× loading buffer (Beyotime, P0286, China) equivalent to one‐quarter of the supernatant volume was added, and the mixture was boiled for 10 min to denature the proteins. The denatured protein samples were separated on 7.5%, 10%, or 12.5% SDS‐PAGE gels (Epizyme, PG112, China) and then transferred onto PVDF membranes (Millipore IPVH00010, USA). The membranes were blocked at room temperature for 30 min and then incubated overnight at 4°C with primary antibodies including Lamin B1 (1:1000, Abcam, ab133741, UK), COL1 (1:3000, Proteintech‐cn, 14695‐1‐AP, USA), MMP9 (1:1000, MCE, HY‐P80756, USA), PINK1 (1:1000, Abmart, PK05715S, China), Parkin (1:1000, Abmart, T56641S, China), HSP60 (1:10000, Proteintech‐cn, 15282‐1‐AP, USA), TIM23 (1:10000, Proteintech‐cn, 11123‐1‐AP, USA), TOM20 (1:10000, Proteintech‐cn, 11802‐1‐AP, USA), P62 (1:1000, CST, 5114), LC3B (1:1000, Proteintech‐cn, 18725‐1‐AP, USA), cGAS (1:1000; Selleck, F045901, USA), p‐STING (1:1000, Abmart, TA7416, China), STING (1:1000, CST, D2P2F, USA), p‐TBK1 (1:1000, CST, D52C2, USA), TBK1 (1:5000, CST, E8I3G, USA), p‐IRF3 (1:1000, CST, D6O1M, USA), IRF3 (1:1000, CST, D83B9, USA), p‐IKK (1:1000, Abmart, TA3014S, China), IKK (1:1000, Abmart, T55660S, China). The next day, the membranes were washed three times with TBST, incubated with HRP‐conjugated goat anti‐mouse IgG (1:5000, CWBIO, CW0102S, China) or HRP‐conjugated anti‐rabbit IgG (1:5000, CWBIO, CW0103S, China) secondary antibody at room temperature for 1 h, and then washed again three times with TBST. Finally, the protein bands were detected using a highly sensitive ECL chemiluminescence reagent (Meilunbio, MA0186‐1, China) and analyzed with ImageJ software.

### 4.9 RT‐qPCR Analysis

FastPure Cell/Tissue Total RNA Isolation Kit V2 (Vazyme, RC112, China) was used to extract total RNA from mouse skin tissue and HaCaT cells according to the manufacturer's instructions. The absorbance values at 260 and 280 nm were measured using a spectrophotometer (Molecular Devices, San Jose, CA, USA) to determine the concentration and purity of total RNA. A reverse transcription kit (Vazyme, R323, China) was used to reverse transcribe total RNA into cDNA, and a SYBR Green kit (Vazyme, Q712, China) was used to perform quantitative RT‐qPCR. After normalization to β‐actin gene expression, relative gene expression was analyzed by the 2^−ΔΔCT^ method. Primer sequences used in this study are listed in Table S1.

### 4.10 Enzyme‐Linked Immunosorbent Assay (ELISA)

The protein abundance of IL‐6 (JL19289, China), IL‐8 (JL19291, China), and IFN‐β (JL19215, China) in the cell supernatant and IL‐6 (JL20268, China) and IFN‐β (JL20219, China) in the serum of blood samples was determined with the indicated ELISA kit (Shanghai Jianglai Biotechnology, China) according to the manufacturer's instructions.

### 4.11 Induction and Treatment of Cell Photoaging

HaCaT cells were cultured in high‐glucose Dulbecco's Modified Eagle Medium (DMEM) (Gibco, USA) supplemented with 10% fetal bovine serum (FBS) (Gibco, USA) and 1% penicillin/streptomycin (Beyotime, C0222, China). at 37°C in a 5% CO_2_ atmosphere.

Upon reaching approximately 50% confluence, the culture medium was aspirated, and the cells were irradiated with UVB. The irradiation protocol consisted of two rounds: an initial exposure at doses of 0, 30, 30, 40, or 40 mJ/cm^2^, followed 24 h later by a second exposure at 0, 30, 40, 40, or 50 mJ/cm^2^, respectively. After irradiation, the cells were treated with the mitophagy agonist CCCP (MCE, HY‐1009441, USA), mitophagy inhibitor Mdivi‐1 (MCE, HY‐15886, USA), STING agonist MSA‐2 (MCE, HY‐136927, USA), and STING inhibitor H151 (MCE, HY‐112693, USA), and the cells were incubated in serum‐free medium with or without SCT for an additional 48 h.

### 4.12 CCK‐8 Assay

HaCaT cells were seeded into 96‐well plates at a density of 5 × 10^3^ cells per well and cultured overnight. After the indicated treatments, 10 μL of CCK‐8 solution (Abbkine, KTA1020, China) was added to each well and incubated at 37°C for 2 h. The optical density was measured at 450 nm using a microplate reader. Cell viability was calculated as the percentage relative to the control group.

### 4.13 SA‐β‐Gal Staining

HaCaT cells were seeded into 6‐well plates and divided into control, UVB, UVB + SCT, and SCT groups. After modeling, the original culture medium was discarded, and the cells were washed three times with PBS for 3 min each. Subsequently, 1 mL of β‐galactosidase staining fixative (Beyotime, C0602, China) was added to each well to fix the cells at room temperature for 15 min. The fixative was then removed, and the cells were washed again with PBS three times for 3 min each. Finally, the staining working solution was added according to the manufacturer's instructions, the plate was sealed with parafilm to prevent evaporation, and incubated overnight at 37°C. The next day, the cells were observed under an optical microscope, and the percentage of SA‐β‐gal‐positive cells was calculated using ImageJ software.

### 4.14 EdU Assay

HaCaT cell proliferation was assessed using an EdU assay kit (RiboBio, C10310‐3, China). Briefly, cells were incubated with an equal volume of EdU working solution in culture medium at 37°C for 2 h. After incubation, samples were fixed with 4% paraformaldehyde, washed, and permeabilized. The Click‐iT reaction mixture was then applied and incubated for 30 min, followed by nuclear staining with Hoechst. Finally, cells were visualized under a fluorescence microscope.

### 4.15 Migration Assay

HaCaT cells were seeded in six‐well plates and incubated at 37 C until a confluent monolayer formed. A standardized wound was created by scraping the monolayer with a p200 pipette tip, followed by washing with PBS to remove dislodged cells. Images of the wound areas were captured at 0, 24, and 48 h post‐scratching. The remaining wound width at predetermined points was measured using ImageJ software. The migration rate was calculated as the ratio of the closed area to the initial wound area.

### 4.16 Immunofluorescence Staining

Skin tissues or cells were fixed with 4% paraformaldehyde (PFA) for 15 min, permeabilized with 0.2% Triton X‐100 in PBS for 10 min, and blocked at room temperature for 1 h. After blocking, the samples were incubated at 4°C overnight with primary antibodies including Lamin B1 (1:300, Abcam, ab133741, UK), HSP60 (1:250, Proteintech‐cn, 15282‐1‐AP, USA), TIM23 (1:500, Proteintech‐cn, 11123‐1‐AP, USA), TOM20 (1:250, Proteintech‐cn, 11802‐1‐AP, USA), followed by PBS washes and subsequent incubation with appropriate secondary antibodies in the dark at room temperature for 1 h. Nuclei were stained with DAPI for 10 min, washed three times with PBS, and then visualized and imaged using a laser scanning confocal microscope.

### 4.17 Colocalization of Mitochondria and Lysosomes

To evaluate mitophagy, mitochondrial–lysosomal colocalization was assessed using two complementary staining approaches. For conventional staining, HaCaT cells were incubated with MitoTracker Red (1:1000, Beyotime, China) at 37°C for 30 min and LysoTracker Green (1:500, Beyotime, China) at 37°C for 1 h, followed by DAPI staining for 10 min. For the second approach, cells were stained with PK Mito Deep Red (1:1000, GenVivo, PKMDR‐2, China) and LysoBrite Red (1:100, AAT Bioquest, 22,645, USA) at 37°C for 30 min and 1 h, respectively, with Hoechst 33,342 (1:100, Beyotime, C1028, China) for nuclear counterstaining. Fluorescence images were acquired using a laser scanning confocal microscope. Colocalization was quantified using ImageJ software with the Coloc 2 plugin, and both Pearson's correlation coefficient and Manders' coefficient were calculated. In addition, Manders' overlap coefficient M1 was calculated, where M1 represents the fraction of mitochondrial fluorescence (channel 1, PK Mito Deep Red) that overlaps with lysosomal signal (channel 2, LysoBrite Red).

### 4.18 Detection of Cytosolic mtDNA Leakage

HaCaT cells were seeded in confocal dishes and subjected to the indicated treatments. Cells were then stained with PicoGreen (1:2000, Invitrogen, P11495, USA), a double‐stranded DNA probe, and PK Mito Deep Red (1:1000, GenVivo, PKMDR‐2, China) at 37°C for 30 min. Nuclei were counterstained with Hoechst 33,342 (1:100, Beyotime, C1028, China). Fluorescence images were acquired using a laser scanning confocal microscope. The fluorescence intensity of cytoplasmic dsDNA and Pearson's correlation coefficient between PicoGreen and PK Mito Deep Red signals were quantified using ImageJ software to evaluate mtDNA release into the cytosol.

### 4.19 Statistical Analyses

Data were expressed as mean ± standard deviation (SD), and all statistical analyses were performed using GraphPad Prism 9.5 software. Intergroup comparisons were analyzed by one‐way ANOVA followed by Tukey's multiple comparison test. Statistical significance was set at *p < 0.05, **p < 0.01, ***p < 0.001, and ****p < 0.0001.

## Author Contributions

T.T.: conceptualization, methodology, investigation, data curation, formal analysis, writing – original draft. M.L., J.Y., X.Y., X.X., X.W., Y.Z., Q.C., S.Z., C.G. and H.Z.: formal analysis and investigation. M.Z., L.Z., and X.W.:conceptualization, supervision, project administration, funding acquisition, writing – review and editing. All authors have read and approved the final version of the manuscript.

## Funding

This work was supported by the National Natural Science Foundation of China (Grant Nos. 82073417, 81703147, and 81402613) and the Guangdong Basic and Applied Basic Research Foundation (Grant No. 2022A1515010768).

## Conflicts of Interest

The authors declare no conflicts of interest.

## Supporting information


**Figure S1:** SCT treatment reverses UV irradiation‐triggered disruption of dermal collagen homeostasis in mouse dorsal skin. (A) Representative images of Masson's trichrome staining in Control, SCT, UV and UV + SCT groups (scale bar, 100 μm). (B) Quantitative analysis of collagen‐positive area from Masson staining, *n* = 3. (C, D) Immunohistochemical staining and statistics of relative levels of COL1, COL3, MMP1, MMP3 and MMP9, *n* = 3 (scale bar, 100 μm). *p < 0.05, **p < 0.01, ***p < 0.001, and ****p < 0.0001.
**Figure S2:** Topical SCT treatment improves basal skin barrier function without altering epidermal and dermal thickness in non‐irradiated mouse skin. (A) Skin elasticity, *n* = 6. (B) Epidermal hydration, *n* = 6. (C) Skin barrier function was detected by transepidermal water loss (TEWL), *n* = 6. (D) Representative images of HE staining from Control and SCT‐only groups (scale bar, 100 μm). (E) Epidermal thickness analysis, *n* = 6. (F) Dermal thickness analysis, *n* = 6. *p < 0.05, **p < 0.01, ***p < 0.001, and ****p < 0.0001.
**Figure S3:** SCT alleviates UV‐triggered epidermal cellular senescence by suppressing the expression of canonical senescence biomarkers p21 and p16. (A) Immunohistochemical staining and statistics of relative levels of p21 and p16 from Control, UV and UV + SCT groups, *n* = 3 (scale bar, 100 μm). *p < 0.05, **p < 0.01, ***p < 0.001, and ****p < 0.0001.
**Figure S4:** SCT normalizes UV‐induced upregulation of PINK1 and Parkin in mouse skin tissue. (A–C) Western blotting was used to measure PINK1 and Parkin expression in skin tissues, *n* = 3. *p < 0.05, **p < 0.01, ***p < 0.001, and ****p < 0.0001.
**Figure S5:** SCT elevates PINK1 and Parkin protein levels and promotes mitochondrial–lysosomal colocalization in UVB‐challenged HaCaT keratinocytes. (A–C) Western blotting was used to measure PINK1 and Parkin expression in HaCaT cells, *n* = 3. (D) Mitochondrial probe and lysosome probe staining in HaCaT cells (scale bar, 10 μm). (E–H) Analysis of the colocalization of the mitochondrial probe and lysosomal probe. *p < 0.05, **p < 0.01, ***p < 0.001, and ****p < 0.0001.
**Figure S6:** SCT exerts its protective effects by enhancing mitophagy. (A) CCK‐8 assay evaluating HaCaT cell viability under various CCCP concentrations, *n* = 3. (B) CCK‐8 assay evaluating HaCaT cell viability under various Mdivi‐1 concentrations, *n* = 3. (C, D) Western blotting was used to measure Lamin B1 expression in HaCaT cells, *n* = 3. *p < 0.05, **p < 0.01, ***p < 0.001, and ****p < 0.0001.
**Figure S7:** SCT reduces UVB‐induced cytosolic dsDNA accumulation in HaCaT cells. (A) Representative confocal microscopy images of HaCaT cells stained with PicoGreen (green, cytosolic dsDNA) and PK Mito Deep Red (red, mitochondria) in the Control, UVB, UVB + SCT, and SCT groups (scale bar, 10 μm). Magnified views (insets) show detailed colocalization of cytosolic dsDNA signals. (B) Quantification of cytoplasmic dsDNA fluorescence intensity in each group. *p < 0.05, **p < 0.01, ***p < 0.001, and ****p < 0.0001.
**Figure S8:** SCT exerts its protective effects by inhibiting the cGAS/STING signaling pathway. (A) CCK‐8 assay evaluating HaCaT cell viability under various MSA‐2 concentrations, *n* = 3. (B) CCK‐8 assay evaluating HaCaT cell viability under various H151 concentrations, *n* = 3. (C, D) Western blotting was used to measure Lamin B1 expression in HaCaT cells, *n* = 3. *p < 0.05, **p < 0.01, ***p < 0.001, and ****p < 0.0001.
**Figure S9:** Mitophagy inhibits UVB‐induced cGAS‐STING pathway activation and inflammatory cytokine production. (A–J) Western blotting was used to measure cGAS, p‐STING, STING, p‐TBK1, TBK1, p‐IRF3, IRF3, p‐IKK, and IKK expression in HaCaT cells, *n* = 3. (K–M) Elisa was performed to measure the concentrations of IL‐6, IL‐8 and IFN‐β in HaCaT cell culture supernatants, *n* = 3. (N–P) Elisa was performed to measure the concentrations of IL‐6, IL‐8 and IFN‐β in HaCaT cell culture supernatants, *n* = 3. *p < 0.05, **p < 0.01, ***p < 0.001, and ****p < 0.0001.
**Figure S10:** SCT alleviates photoaging by enhancing mitophagy to suppress cGAS‐STING pathway activation and inflammatory responses. (A–F) Western blotting was used to measure HSP60, TIM23, TOM20, p62 and LC3B‐II expression in HaCaT cells, *n* = 3. (G–P) Western blotting was used to measure cGAS, p‐STING, STING, p‐TBK1, TBK1, p‐IRF3, IRF3, p‐IKK, and IKK expression in HaCaT cells, *n* = 3. *p < 0.05, **p < 0.01, ***p < 0.001, and ****p < 0.0001.
**Figure S11:** SCT ameliorates photoaging via the mitophagy‐cGAS‐STING axis. (A–C) Elisa was performed to measure the concentrations of IL‐6, IL‐8 and IFN‐β in HaCaT cell culture supernatants, *n* = 3. (D, E) Western blotting was used to measure Lamin B1 expression in HaCaT cells, *n* = 3. *p < 0.05, **p < 0.01, ***p < 0.001, and ****p < 0.0001.
**Figure S12:** SCT modulates the mitophagy–cGAS‑STING axis and alleviates photoaging in vivo. Skin tissue and serum samples were collected from mice in four experimental groups: UV + SCT, UV + SCT + Mdivi‑1, UV + SCT + H151, and UV + SCT + Mdivi‑1 + H151. (A–D) Western blotting was used to measure PINK1, Parkin, and HSP60 expression in skin tissues, *n* = 3. (E–H) Western blotting was used to measure cGAS, p‐STING and STING expression in skin tissues, *n* = 3. (I, J) Elisa was performed to measure the concentrations of IL‐6 and IFN‐β in mouse serum, *n* = 3. (K, L) Western blotting was used to measure Lamin B1 expression in skin tissues, *n* = 3. *p < 0.05, **p < 0.01, ***p < 0.001, and ****p < 0.0001.
**Table S1:** Primer sequences used in the study.

## Data Availability

The data that supports the findings of this study are available in the Supporting Information of this article.
