# Supplementary material for: The Secretome Derived From Human Umbilical Cord Mesenchymal Stem Cells Improves Skin Photoaging by Enhancing Mitophagy to Inhibit the cGAS‐STING Pathway
Source: Aging Cell. 2026 Sep 11;25(9):e70701. doi: 10.1111/acel.70701 (PMC13563172; doi:10.1111/acel.70701)
Supplement: Supplementary file 1 [file ACEL-25-e70701-s001.docx]

**Supplementary Information**

**The secretome derived from human umbilical cord mesenchymal stem cells improves skin photoaging by enhancing mitophagy to inhibit the cGAS-STING pathway**

**This file includes:**

Supplementary Figures 1 to 12

Supplementary Table 1

**Supplementary** **Figure 1. SCT treatment reverses UV irradiation-triggered disruption of dermal collagen homeostasis in mouse dorsal skin.** (A) Representative images of Masson’s trichrome staining in Control, SCT, UV and UV+SCT groups (scale bar, 100 μm). (B) Quantitative analysis of collagen-positive area from Masson staining, n = 3. (C,D) Immunohistochemical staining and statistics of relative levels of COL1, COL3, MMP1, MMP3 and MMP9, n = 3 (scale bar, 100 μm). *P < 0.05, **P < 0.01, ***P < 0.001, and ****P < 0.0001.

**Supplementary** **Figure 2. Topical SCT treatment improves basal skin barrier function without altering epidermal and dermal thickness in non-irradiated mouse skin.** (A) Skin elasticity, n = 6. (B) Epidermal hydration, n = 6. (C) Skin barrier function was detected by transepidermal water loss (TEWL), n = 6. (D) Representative images of HE staining from Control and SCT-only groups (scale bar, 100 μm). (E) Epidermal thickness analysis, n = 6. (F) Dermal thickness analysis, n = 6. *P < 0.05, **P < 0.01, ***P < 0.001, and ****P < 0.0001.

**Supplementary Figure 3. SCT alleviates UV-triggered epidermal cellular senescence by suppressing the expression of canonical senescence biomarkers p21 and p16.** (A) Immunohistochemical staining and statistics of relative levels of p21 and p16 from Control, UV and UV+SCT groups, n = 3 (scale bar, 100 μm). *P < 0.05, **P < 0.01, ***P < 0.001, and ****P < 0.0001.

**Supplementary Figure 4. SCT normalizes UV-induced upregulation of PINK1 and Parkin in mouse skin tissue.** (A-C) Western blotting was used to measure PINK1 and Parkin expression in skin tissues, n = 3. *P < 0.05, **P < 0.01, ***P < 0.001, and ****P < 0.0001.

**Supplementary Figure 5. SCT elevates PINK1 and Parkin protein levels and promotes mitochondrial–lysosomal colocalization in UVB-challenged HaCaT keratinocytes.** (A-C) Western blotting was used to measure PINK1 and Parkin expression in HaCaT cells, n = 3. (D) Mitochondrial probe and lysosome probe staining in HaCaT cells (scale bar, 10 μm). (E-H) Analysis of the colocalization of the mitochondrial probe and lysosomal probe. *P < 0.05, **P < 0.01, ***P < 0.001, and ****P < 0.0001.

**Supplementary** **Figure 6. SCT exerts its protective effects by enhancing mitophagy.** (A) CCK-8 assay evaluating HaCaT cell viability under various CCCP concentrations, n = 3. (B) CCK-8 assay evaluating HaCaT cell viability under various Mdivi-1 concentrations, n = 3. (C,D) Western blotting was used to measure Lamin B1 expression in HaCaT cells, n = 3. *P < 0.05, **P < 0.01, ***P < 0.001, and ****P < 0.0001.

**Supplementary** **Figure 7. SCT reduces UVB-induced cytosolic dsDNA accumulation in HaCaT cells.** (A) Representative confocal microscopy images of HaCaT cells stained with PicoGreen (green, cytosolic dsDNA) and PK Mito Deep Red (red, mitochondria) in the Control, UVB, UVB+SCT, and SCT groups (scale bar, 10 μm). Magnified views (insets) show detailed colocalization of cytosolic dsDNA signals. (B) Quantification of cytoplasmic dsDNA fluorescence intensity in each group. *P < 0.05, **P < 0.01, ***P < 0.001, and ****P < 0.0001.

**Supplementary** **Figure 8. SCT exerts its protective effects by inhibiting the cGAS/STING signaling pathway.** (A) CCK-8 assay evaluating HaCaT cell viability under various MSA-2 concentrations, n = 3. (B) CCK-8 assay evaluating HaCaT cell viability under various H151 concentrations, n = 3. (C,D) Western blotting was used to measure Lamin B1 expression in HaCaT cells, n = 3. *P < 0.05, **P < 0.01, ***P < 0.001, and ****P < 0.0001.

**Supplementary** **Figure 9. Mitophagy inhibits UVB-induced cGAS-STING pathway activation and inflammatory cytokine production. (**A-J) Western blotting was used to measure cGAS, p-STING, STING, p-TBK1, TBK1, p-IRF3, IRF3, p-IKK and IKK expression in HaCaT cells, n = 3. (K-M) ELISA was performed to measure the concentrations of IL-6, IL-8, and IFN-β in HaCaT cell culture supernatants, n = 3. (N-P) ELISA was performed to measure the concentrations of IL-6, IL-8, and IFN-β in HaCaT cell culture supernatants, n = 3. *P < 0.05, **P < 0.01, ***P < 0.001, and ****P < 0.0001.

**Supplementary** **Figure 10. SCT alleviates photoaging by enhancing mitophagy to suppress cGAS-STING pathway activation and inflammatory responses.** (A-F) Western blotting was used to measure HSP60, TIM23, TOM20, p62 and LC3B-II expression in HaCaT cells, n = 3. **(**G-P) Western blotting was used to measure cGAS, p-STING, STING, p-TBK1, TBK1, p-IRF3, IRF3, p-IKK and IKK expression in HaCaT cells, n = 3. *P < 0.05, **P < 0.01, ***P < 0.001, and ****P < 0.0001.

**Supplementary** **Figure 11. SCT ameliorates photoaging via the mitophagy-cGAS-STING axis.** (A-C) ELISA was performed to measure the concentrations of IL-6, IL-8, and IFN-β in HaCaT cell culture supernatants, n = 3. (D,E) Western blotting was used to measure Lamin B1 expression in HaCaT cells, n = 3. *P < 0.05, **P < 0.01, ***P < 0.001, and ****P < 0.0001.

**Supplementary** **Figure 12. SCT modulates the mitophagy–cGAS‑STING axis and alleviates photoaging in vivo.** Skin tissue and serum samples were collected from mice in four experimental groups: UV+SCT, UV+SCT+Mdivi‑1, UV+SCT+H151, and UV+SCT+Mdivi‑1+H151. (A-D) Western blotting was used to measure PINK1, Parkin, and HSP60 expression in skin tissues, n = 3. (E-H) Western blotting was used to measure cGAS, p-STING and STING expression in skin tissues, n = 3. (I, J) ELISA was performed to measure the concentrations of IL-6 and IFN-β in mouse serum, n = 3. (K, L) Western blotting was used to measure Lamin B1 expression in skin tissues, n = 3. *P < 0.05, **P < 0.01, ***P < 0.001, and ****P < 0.0001.

**Supplementary Table 1. Primer sequences used in the study.**

| Primer name | Species |  | Primer sequences |
| --- | --- | --- | --- |
| β-actin | Mus musculus | Forward | GGCTGTATTCCCCTCCATCG |
|  |  | Reverse | CCAGTTGGTAACAATGCCATGT |
| IL-6 |  | Forward | GGGACTGATGCTGGTGACAA |
|  |  | Reverse | ACAGGTCTGTTGGGAGTGGT |
| IFN-β |  | Forward | CAGCTCCAAGAAAGGACGAAC |
|  |  | Reverse | GGCAGTGTAACTCTTCTGCAT |
| Actin | Homo sapiens | Forward | CATGTACGTTGCTATCCAGGC |
|  |  | Reverse | CTCCTTAATGTCACGCACGAT |
| IL-6 |  | Forward | ACTCACCTCTTCAGAACGAATTG |
|  |  | Reverse | CCATCTTTGGAAGGTTCAGGTTG |
| IL-8 |  | Forward | CTGGCCGTGGCTCTCTTG |
|  |  | Reverse | CCTTGGCAAAACTGCACCTT |
| IFN-β |  | Forward | AGTGTCAGAAGCTCCTGTGG |
|  |  | Reverse | CATAGATGGTCAATGCGGCG |
